# Corneal neuropathic pain: a review to inform clinical practice

**DOI:** 10.1038/s41433-024-03060-x

**Published:** 2024-04-16

**Authors:** Stephanie L. Watson, Damien Tuan-Man Le

**Affiliations:** 1 The University of Sydney, Save Sight Institute, Faculty of Medicine and Health, Sydney, NSW Australia; 2 Sydney Eye Hospital, Sydney, NSW Australia

**Keywords:** Drug therapy, Corneal diseases

## Abstract

Corneal neuropathic pain (CNP) is a poorly defined disease entity characterised by an aberrant pain response to normally non-painful stimuli and categorised into having peripheral and central mechanisms, with the former responding to instillation of topical anaesthetic. CNP is a challenging condition to diagnose due to numerous aetiologies, an absence of clinical signs and ancillary tests (in vivo confocal microscopy and esthesiometry), lacking the ability to confirm the diagnosis and having limited availability. Symptomatology maybe mirrored by severe and chronic forms of dry eye disease (DED), often leading to misdiagnosis and inadequate treatment. In practice, patients with suspected CNP can be assessed with questionnaires to elicit symptoms. A thorough ocular assessment is also performed to exclude any co-existent ocular conditions. A medical and mental health history should be sought due to associations with autoimmune disease, chronic pain syndromes, anxiety and depression. Management begins with communicating to the patient the nature of their condition. Ophthalmologists can prescribe topical therapies such as autologous serum eyedrops to optimise the ocular surface and promote neural regeneration. However, a multi-disciplinary treatment approach is often required, including mental health support, particularly when there are central mechanisms. General practitioners, pain specialists, neurologists and psychologists may be needed to assist with oral and behavioural therapies. Less data is available to support the safety and efficacy of adjuvant and surgical therapies and the long-term natural history remains to be determined. Hence clinical trials and registry studies are urgently needed to fill these data gaps with the aim to improve patient care.

## Abstract (zh)

角膜神经痛 (CNP) 是一种定义不清的疾病, 其特征是对正常非痛性刺激出现异常疼痛反应, 可分为外周和中枢机制, 前者对表麻有反应。由于病因众多、缺少临床症状以及辅助检查 (体内共聚焦显微镜检查和触觉测量), CNP的诊断具有挑战性, 不仅无法确诊, 且可行性有限。症状可能表现为严重和慢性干眼病 (DED), 经常导致误诊治疗不足。在实际中, 可以使用问卷调查来评估疑似CNP的患者。进行彻底的眼部评估以排除任何共存的眼部疾病。由于其与自身免疫性疾病、慢性疼痛综合征、焦虑和抑郁有关, 应问诊病史和心理健康史。治疗首先要与患者说明其病情的性质。眼科医生可以开具局部疗法, 如自体血清眼药水, 以优化眼表并促进神经再生。然而, 通常需要采取多学科治疗, 包括心理健康支持, 特别是当存在中枢机制时。可能需要全科医生、疼痛专家、神经学家和心理学家协助口头和行为疗法。支持辅助疗法和手术疗法的安全性和有效性的数据较少, 长期自然史尚有待确定。因此, 迫切需要临床试验和注册研究以填补这些数据空白, 以改善患者护理。

## Introduction

Corneal neuropathic pain (CNP) is being increasingly recognised, particularly in patients with a diagnosis of dry eye disease (DED) [1], for its impact on a patient’s quality of life [2, 3]. The impacts can be mild with minimal effects on activities of daily living to severe with the patient experiencing debilitating symptoms that can lead to a deterioration in their physical and social well-being [4]. Reports have emerged of its occurrence and burden following cataract and refractive surgery [5–7] and in those with neurotrophic keratopathy [8], chronic pain syndromes [9, 10] and autoimmune diseases [11, 12]. The overarching feature of CNP is a heightened experience of pain without commensurate clinical signs [4, 13, 14]. Other terminology used to describe the condition include ocular neuropathic pain, corneal neuralgia, neuropathic corneal pain, ocular pain syndrome, corneal pain syndrome, keratoneuralgia, corneal neuropathic disease, phantom cornea, corneal neuropathy and corneal allodynia [13]. Central and peripheral CNP have been defined, with the former responding to topical analgesia [15]. Subtypes of CNP have been identified as; associated with (1) specific ocular disease (2) ocular surface disease without keratitis (3) systemic pain syndrome (4) psychiatric disease (especially depression) and (5) idiopathic [16]. More recently, a new disease association has been noted between CNP and Long COVID [17].

A range of symptoms can be produced by CNP with many overlapping those of DED, such that it is frequently misdiagnosed as dry eye [13, 18]. With corneal vital dye staining - a mainstay in identifying ocular surface damage for the diagnosis of DED [19], CNP has been referred to as ‘pain without stain’. It has been proposed that CNP may be a subtype of Sjogren International Collaborative Clinical Alliance (SICCA) dry eye [20], such that CNP may be the extreme end of the dry eye spectrum [3]. Increased pain sensitivity may influence perceptions of ocular discomfort and dryness and has been reported by contact lens wearers [21].

There remains a lack of epidemiological, long-term and high-quality clinical trial data on CNP, and many clinicians are unfamiliar with the existence of the condition and how to manage it. Registry data is lacking and needed to provide long-term outcomes and disease natural history [22]. Further, current diagnosis of CNP is by exclusion as there is no ‘gold standard’ and patient phenotypes are poorly understood. Evidence is emerging on tools for diagnosis including questionnaires. Technologies such as in vivo confocal microscopy (IVCM) and esthesiometry, have been used to support a diagnosis of CNP but their utility in everyday clinical practice is unknown [15, 23]. Limited clinical studies and trials have provided some data on potential topical, oral, adjuvant and surgical therapies for CNP. Further research is needed to inform the development of evidence-based guidelines for the diagnosis and management of CNP [14].

### What is the underlying pathophysiology of corneal neuropathic pain?

The International Association for the Study of Pain defined neuropathic pain as ‘pain caused by a lesion or disease of the somatosensory system’ [4, 24]. CNP can be considered a part of this disorder as it is associated with injury to the corneal nerves, terminal endings of the ophthalmic division of the trigeminal somatosensory system [1, 4, 13, 14]. Corneal nerves can be damaged by a variety of peripheral and systemic aetiologies [1, 4, 13, 14]. Peripheral nerve injuries can result from ocular surface diseases such as DED, contact lens wear, infections, surgery, trauma, toxins, and radiation [1, 4, 13, 14]. In comparison, systemic diseases damage corneal nerves through chronic inflammation and can include disorders such as Systemic Lupus Erythematosus, sarcoidosis, fibromyalgia, diabetes and small-fibre polyneuropathies [1, 4, 13, 14]. In CNP there are two neurobiological processes—peripheral and central sensitisation [1, 14]. Sensitisation can occur after an initial insult with sub-threshold noxious stimuli (hyperalgesia) [4, 13, 14, 18, 25] or even non-noxious stimuli (allodynia, photoallodynia) [18, 26, 27]. Genetic factors may likely contribute to the occurrence of CNP. A variety of genetic polymorphisms have been identified on genome wide association studies in a cohort of veterans CNP [28]. The protein products of the implicated genes may have a role in sensory perception and potentially have links to DED [28].

Peripheral sensitisation occurs when injury to peripheral axons results in the release of pro-inflammatory mediators such as cytokines, prostaglandins and substance P, which decrease the threshold potentials of nociceptors, leading the axons to be triggered by previously non-painful stimuli [1, 4, 13, 14]. Over time, increased peripheral sensitisation results in central neurons becoming highly responsive to non-painful stimuli, leading to an increased response to overall pain, known as central sensitisation [1, 4, 13, 14]. Central CNP is due to abnormal function of the pain cortex in the brain reacting to stimuli that are unpleasant or noxious. Whereas in peripheral CNP, the peripheral sensory nerves are overly sensitive and respond to stimuli that are subthreshold (allodynia), light/non-noxious (photoallodynia) or suprathreshold (hyperalgesia) [15]. These neurobiological processes ultimately produce a wide range of symptoms including hyperalgesia, allodynia, photoallodynia, itching, irritation, burning, dryness, foreign body sensation and a feeling of pressure [1, 4, 13, 14]. Further, neuropathic ocular itch and pain can occur together; with both a result of ocular surface nerve damage and dysfunction [29]. Underlying itch and pain is likely due to inflammation and immune system upregulation [29].

#### Implications for practice

- CNP is a subtype of neuropathic pain arising from damage to the corneal nerves.
- Stimuli that usually do not elicit pain may produce CNP.
- Symptoms of CNP include itching, irritation, burning, dryness and foreign body sensation along with feelings of pressure.

### Who gets corneal neuropathic pain?

CNP may be associated with systemic diseases, with a higher prevalence of females affected, such as autoimmune conditions and fibromyalgia [1, 4, 13, 14]. CNP may also occur with other ocular conditions or following trauma or surgery (e.g. cataract and refractive surgery). Associated ocular conditions include DED, infectious keratitis, herpes simplex keratitis, herpes zoster ophthalmicus, recurrent corneal erosion, radiation keratopathy [4, 14, 25, 30]. Refractive surgery has been known to induce DED [31] but is increasingly being reported to induce ocular pain [4, 6, 14, 25, 30]. Patients with CNP due to refractive surgery and herpes simplex keratitis may have similar clinical characteristics and report moderate to severe pain levels [7]. Both conditions have moderate impacts on quality of life and a significant reduction in total nerve density compared to healthy controls on IVCM [7].

#### High index of suspicion for CNP

- A history of ocular and/or systemic disease should be sought in patients who are suspected to have CNP.
- Persistent pain following ocular surgery or infection should raise a high index of suspicion for CNP.

### Dry eye disease and corneal neuropathic pain

DED and CNP may occur in the same patient and CNP can exacerbate the symptoms of DED. Indeed, CNP has been associated with more severe dry eye symptoms in an ophthalmology clinic patient population [9]. In DED, there may be various causes of ocular surface damage including infection, inflammation, trauma, adverse environmental conditions, abnormal ocular anatomy and high tear osmolarity [1, 25]. If this damage persists, or if the vicious cycle of DED is not broken, peripheral and central sensitisation can occur, leading to neuropathic pain [1, 25]. As such, some patients with DED may report symptoms that are out of proportion to their ocular surface findings including allodynia, hyperalgesia and hyperaesthesia [1, 25]. Indeed, an overlap exists between CNP and more severe and chronic forms of DED [1, 20, 25]. The DEWS TFOS definition of DED includes the role of neurosensory abnormalities in disease aetiology [32] and peripheral CNP is characterised by sensitisation of sensory and/or nociceptive processing at some level of the trigeminal system. One way to distinguish DED from CNP, there should be a therapeutic failure of conventional treatment for DED and a lack of therapeutic response to topical anaesthetics if the CNP is peripheral [1]. In peripheral neuropathic pain, there may be cutaneous allodynia i.e. pain to light touch around the eye [29]. Indeed as the presence of neuropathic pain is not routinely sought in dry eye patients [25], it has been proposed that such patients should be screened for CNP [33].

#### Implications for practice

- In chronic DED, particularly if it is severe, if pain is out of proportion to the clinical signs, particularly with associated allodynia, consider CNP.
- When the patient fails standard dry eye therapy, consider CNP.

### Diagnosis of corneal neuropathic pain

There are no standardised diagnostic criteria for CNP. Further, the variability in CNP symptoms often makes it challenging to establish a diagnosis, especially considering the significant overlap with DED, and the lack of clinical signs on examination [1, 4, 13, 14]. Characteristic symptoms include pain, dryness, and itch along with burning, sensitivity to wind, light and temperature [9]. Patients may report of indistinct sensations of pressure [4] and episodes of spontaneous pain [25]. Such symptoms can be present in ocular surface diseases including DED [1].

Validated questionnaires can assist in the diagnosis of CNP [16] as the overlap between severe and chronic DED and CNP led to a variety of DED questionnaires being used to screen for and assess the impact on visual function and quality of life (Table 1) [25, 34–41]. However, such questionnaires were specific for DED and not CNP [25] as most DED questionnaires were not able to differentiate between nociceptive and neuropathic symptoms [1, 25].

**Table 1:** Questionnaires that assess corneal neuropathic pain [92].

Category	Questionnaire
Dry Eye Disease	Ocular Surface Disease Index
	Impact of Dry Eye in Everyday Life (IDEEL)
	Dry Eye Quality of Life Score (DEQS)
	Dry Eye Questionnaire (DEQ)
	Dry Eye Questionnaire 5 (DEQ-5)
	Ocular Comfort Index
	Standard Patient Evaluation of Eye Dryness questionnaire (SPEED)
	Symptom Assessment Questionnaire in Dry Eye (SANDE)
	McMonnies Dry Eye Index
	Short Questionnaire for Dry Eye Syndrome
	Modified Schein Dry Eye Questionnaire
	University of North Caroline Dry Eye Management Scale (UNC DEMS)
Blepharitis	Blepharitis Symptom Measure (BLISS)
	Meibomian Gland Dysfunction-Specific Symptom Questionnaire
Refractive Surgery	Patient-Reported Outcomes with LASIK (PROWL)
	National Eye Institute-Refractive Error Quality of Life instrument (NEI-RQL)
Contact Lens	Contact Lens Dry Eye Questionnaire (CLDEQ)
	Contact Lens Dry Eye Questionnaire 8 (CLDEQ-8)
	Contact Lens Discomfort Index (CLDI)
Sjogren’s Syndrome	Liverpool Sicca Index
	EULAR Sjogren’s Syndrome Patient Reported Index (ESSPRI)
	Sicca Symptoms Inventory
Non-specific Ophthalmic	National Eye Institute Vision Function Questionnaire (NEI-VFQ)
	National Eye Institute Vision Function Questionnaire 25 (NEI-VFQ 25)
	National Eye Institute Vision Function Questionnaire 39 (NEI-VFQ 39)
Ocular Pain	Ocular Pain Assessment Survey (OPAS)
	Neuropathic Pain Symptom Inventory – Eye (NPSI-Eye)
	Eye Sensation Scale

Questionnaires specific for ocular pain are now available such as the Ocular Pain Assessment Survey, which is a quantitative, multidimensional questionnaire used to assess corneal and ocular surface pain and the Neuropathic Pain Symptom Inventory—Eye (NPSI-Eye), which is a modified version of the Neuropathic Pain Symptom Inventory (NPSI) that specifically assesses neuropathic-like ocular pain [42, 43]. These questionnaires are valid and reliable, but further studies are needed to validate preliminary findings on their use in CNP [42, 43]. Questionnaires have also been used to assess diseases associated with CNP. For example, contact lens wear maybe a cause of CNP with symptoms of dryness, grittiness, scratchiness and foreign body sensation being identified on questionnaires [44] such as the contact lens dry eye questionnaire and contact lens discomfort index [45–47] (Table 1). In terms of systemic diseases, validated questionnaires such as the Liverpool Sicca index, EULAR Sjogren’s Syndrome Patient Reported Index and Sicca Symptoms Inventory have also been used to evaluate patients with primary Sjogren’s syndrome and include ocular symptoms such as dryness, irritation and poor vision [48–50] (Table 1).

History taking should include details on ophthalmic symptoms and their onset as well as a comprehensive assessment of systemic and mental health. In patients with ocular surface disease and persistent pain despite treatment, a diagnosis of CNP should be suspected [51]. Patients should be specifically asked about a history of chronic pain disorders (migraine, fibromyalgia, traumatic brain injury) and their treatment [52]. All prior ocular surgical procedures, particularly refractive laser surgery and cataract surgery should be documented along with the temporal onset of symptoms in relation to the procedure. Further, patients with CNP should be screened for depression and post-traumatic stress disorder through validated questionnaires such as the Patient Health Questionnaire 9 and post-traumatic stress disorder using the PTSD checklist—Military Version (PCL-M) [33]. This should be a particular consideration in groups such as veterans [33] as an association between pain intensity and mental health has been described [16].

#### Implications for practice

- A comprehensive ophthalmic, general and mental health history is needed to identify causal and associated conditions. Questionnaires are an emerging tool for use in CNP and ocular pain questionnaires can screen for CNP.
- Disease-specific questionnaires can identify associated conditions, for example DED.
- Mental health questionnaires should be used to screen for conditions such as depression and post-traumatic stress disorder.

### Assessment of corneal neuropathic pain

Assessment should begin with evaluation of ocular surface health. Evaluation of the periocular and facial skin along with eyelid function is carried out to identify conditions including rosacea, atopy, blepharitis, ectropion, trichiasis, entropion and a decreased blink rate [52]. Corneal sensation in each eye should be tested and compared, with the clinician looking for increased or decreased responses. Slit lamp examination can be used to assess the health of the ocular surface; with vital dyes (fluorescein, lissamine green, rose Bengal) able to reveal compromise of the corneal and/or conjunctival surface. Other tests that can assess ocular surface health and the presence or absence of co-existent DED include; tests that can evaluate the tear film such as the Schirmer’s test, phenol red test, tear film osmolarity and tear break up time For some people who experience CNP, tests designed to diagnose and characterise DED maybe normal unless there is co-existent DED [4].

Instillation of topical anaesthetic, for example 0.5% proparacaine hydrochloride (Alcaine, Alcon) [4] is helpful in distinguishing peripheral from central pain. The topical anaesthetic will reduce peripheral pain, have no effect in central pain and may have a lesser effect if there is a combination of central and peripheral pain [4]. In our experience, peripheral CNP generally responds to treatment and should be treated early as central sensitisation may follow which is more difficult to treat [53]. Evoked pain to light or reports of photophobia can indicate central sensitisation in patients with CNP [54, 55].

#### Lessons for practice

- Assessment of the ocular surface is needed to diagnose and determine the severity of any underlying disease, in particular DED.
- Topical anaesthetic will reduce peripheral pain allowing it to be distinguished from central CNP.
- Pain to light/photophobia can indicate central CNP.

### Investigations for corneal neuropathic pain

**Fig. 1:**
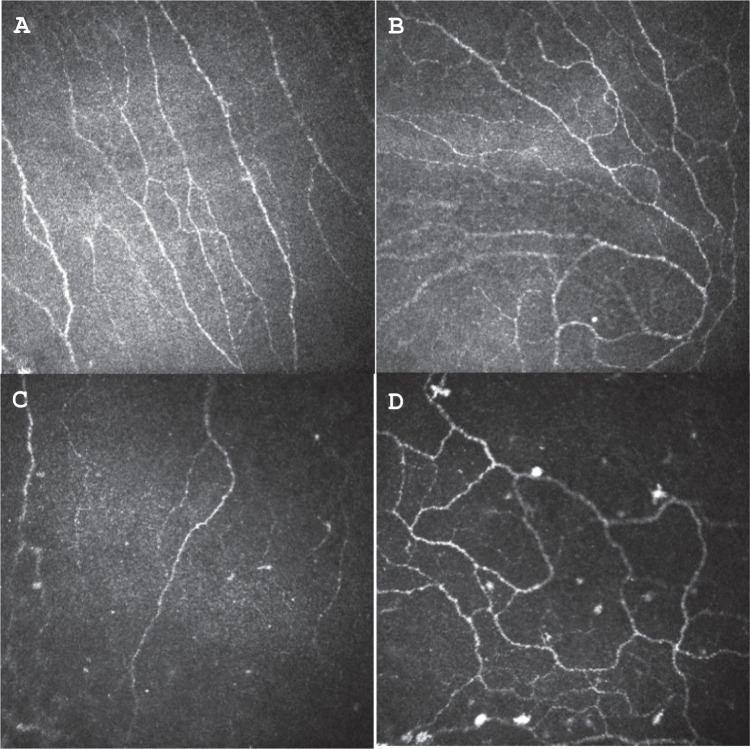
Corneal nerves imaged with In Vivo Confocal Microscopy. **A**, **B** normal corneal nerve patterns. **C**, **D** reduced corneal nerve density in patients with diabetes (Courtesy of Associate Professor Maria Markoulli).

Limited investigations are available to support the diagnosis including in vivo confocal microscopy (IVCM) and esthesiometers. IVCM is a non-invasive imaging technique that has been used in CNP and other conditions to allow the detection of corneal nerve abnormalities, to differentiate the various aetiologies and monitor treatment efficacy [1, 4, 13, 14] (Fig. 1). With IVCM, the corneal nerves and cells and immune cells can be imaged [56, 57]. Alterations in the sub-basal nerve plexus have been found in patients with CNP with IVCM [26] but also in normal subjects as well as those with ocular surface disease including DED [16]. The presence of neuromas and neural sprouting has been described [15] but was not found to be significantly different between the control group and patients with central CNP [16]. Activated keratocytes and spindle, lateral and stump microneuromas have been reported in patients with central and peripheral CNP on IVCM [15]. A potential biomarker may be that a greater number of microneuromas and activated keratocytes are seen with IVCM in patients with CNP that respond to topical anaesthesia [15]. Corneal sensation can also be measured using esthesiometers, such as the Cochet–Bonnet contact device and the noncontact Belmonte ethesiometer[58]. Esthesiometers can detect mechanical nociceptor responses and quantify nerve fibre functionality [1, 4, 13, 14]. Overall, abnormalities in corneal sensitivity and morphology can only suggest and not confirm a diagnosis of CNP [25]. At the time of writing, these tools are not readily available to clinicians in everyday practice.

#### Implications for practice

- Investigations for CNP can provide structural and functional information to aid diagnosis and monitor treatment response but are not essential for the management of CNP.

### Management

**Fig. 2:**
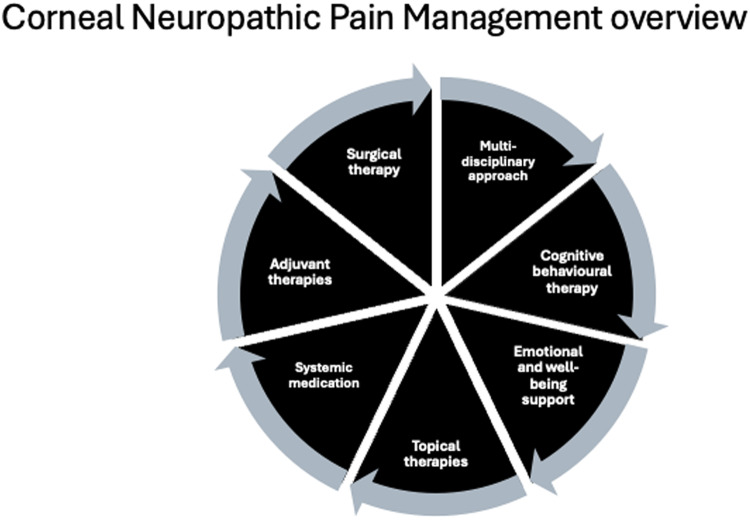
Modalities that have been used to treat corneal neuropathic pain. A multidisciplinary approach is needed with cognitive behavioural therapy, emotional and well-being support, topical therapies, systemic medication, adjuvant therapies and, lastly, surgical therapy available as options.

To date, management of CNP has been guided by evidence-based literature on systemic neuropathic pain as well as post-herpetic neuralgia [14]. Treatment of neuropathic pain is generally complex as several treatment modalities may be needed due to its varied and intricate pathophysiology [53] (Fig. 2). A multi-disciplinary approach can optimise patient care and enable the most suitable mode of treatment to be selected [16]. Topical and systemic medications maybe [7] needed and rarely surgical interventions. Adjunctive and alternative therapies may be considered and are generally chosen based on whether the damage to the somatosensory system is peripheral or central [52].

Treatment of CNP typically begins by removing any inciting factors and/or treating underlying causal or exacerbating disease [29]. Key components of management include the use of anti-inflammatories, agents that may regenerate nerves and addressing any mental health issues [4, 26, 59, 60]. Targeting inflammation is an initial step in management [18] as damage to corneal nerves has been associated with inflammation [61]. An explanation of the condition should be given to patients with reassurance that the cornea has the most potential to produce pain in the body and therefore symptoms can be significant [4]. Patients can be offered cognitive behavioural therapy, emotional support and counselling.

### Topical therapies

Most topical therapies used to manage CNP aim to reduce inflammation and promote the health of the ocular surface and its nerves [52], with the mainstays of therapy being anti-inflammatory agents and autologous serum [62]. A range of additional agents, able to modulate nerve activity and regeneration, are under investigation for use in CNP although evidence is lacking on their efficacy and safety for everyday clinical use (Table 2).

**Table 2:** Topical ophthalmic preparations and their potential mechanism of action in corneal neuropathic pain [33, 69, 75].

Topical therapy	Mechanism of action
Corticosteroids	Anti-inflammatory actions can reduce the dendritic cell density
Autologous serum	Promotes ocular surface health and reduces abnormalities in the sub-basal nerve plexus
Lacosamide	Amino acid molecule that decreases hyperexcitability of cold-sensitive corneal nerve terminals
Low dose naltrexone	Opioid antagonist with effects on systemic neuropathic pain
Enkephalin modulators	Neuropeptide inhibitor modulates pain
Libvatrep	Receptor potential vanilloid 1 antagonist
Topical nerve growth factor	Corneal nerve regeneration

Topical corticosteroids have been proposed to modulate antigen-presenting cells including dendritic cells in both CNP and DED. These cells have an important role in immune cascades and have been implicated in the pathogenesis of both corneal pain and DED [27, 63, 64]. Topical corticosteroids maybe particularly useful in patients where sub-basal dendritic cells have been found on IVCM [65, 66]. Topical cyclosporine has been associated with an improvement in comfort and nerve density in patients with dry eye and chronic ocular surface pain [67, 68]. As such, topical cyclosporine and similarly lifitegrast and tacrolimus may be of benefit as anti-inflammatories for CNP [52].

Autologous serum eyedrops are composed of growth factors, vitamins, albumin and cytokines and are more similar to tears than artificial lubricants [69]. The use of autologous serum is based on reversing the underlying damage that is hypothesised to underlie CNP [26, 62]. In addition, they may have a role in modulating the immune system [52]. Autologous serum eye drops, in a retrospective case series of 16 patients with severe CNP and no active ocular surface disease vs 12 controls, was found to significantly improve pain symptoms with signs of corneal nerve regeneration on IVCM [26]. Their role in DED has not been supported by all studies, with a Cochrane review finding only a trend towards improvement [70]. High-quality clinical trial data is needed to identify the efficacy and safety of autologous serum preparations. Further, preparations such as fresh frozen plasma and platelet-enriched plasma in theory may have benefit for CNP and are awaiting high quality clinical evidence to support their use [66, 71].

### Novel therapies

A range of novel therapies are under investigation for CNP. For example, a topical nerve growth factor has been established as a treatment for neurotrophic keratitis [72]. Topical lacosamide, an aminoacid molecule developed as an anti-epileptic, has been shown in an ex vivo model to decrease hyperexcitable cold-sensitive nerve terminals in the cornea [73]. As cold sensitive nerve terminals have a role in the perception of pain it may have a role in CNP [74]. Lacosamide 1% has been compounded for topical use and has been categorised as a Schedule 5 drug by the Federal Drug Agency in the USA [66]. Topical low dose naltrexone is also under development. Naltrexone is an opioid antagonist that has been given orally for opioid and alcohol addiction that has also been used in low doses for systemic neuropathic pain [75]. As well as antagonising opoid receptors, naltrexone acts on non-opioid receptors such as the Toll-like receptor on macrophages and microglia and has roles in modulating pain. Topical naltrexone may increase corneal healing rates and promote corneal epithelial cell division [76]. In a small phase 1 study topical naltrexone was found to be tolerable in escalating doses in normal volunteers [77]. Low-dose naltrexone can be compounded as an eyedrop but is a high-risk product due to issues with sterility [66] and there is a lack of clinical evidence on its efficacy and safety in CNP. Topical enkephalin modulators may have a therapeutic role via their actions as a neuropeptide inhibitor to modulate pain [66, 76]. Libvatrep (transient receptor potential vanilloid 1 antagonist) a topical TRPV1 antagonist SAF312 (libvetrep) has been investigated for post-surgical pain in a clinical trial including patients following photorefractive keratectomy [78]; it may have a role in CNP.

### Systemic therapy

A range of oral therapies may have a role in the management of CNP (Table 3). Pain specialists or neurologists can contribute to the treatment of CNP as they can have a role in prescribing such drugs for neuropathic pain [16]. For instance, in central neuropathic pain, oral neuromodulators may have some success in relieving the symptoms of CNP [29]. In a case series that included 8 patients, gabapentin was commenced at 300 mg orally daily and increased to 600–900 mg three times a day and pregabalin was commenced at 75 mg daily and increased to 150 mg twice a day. In this trial, the therapy was successful in five patients, produced mild relief in one patient and two patients had no improvement [79]. Combination therapy with serotonin norepinephrine reuptake inhibitors was also used in the study [79], and in patients with DED and CNP a significant improvement in pain symptoms as well as dry eye scores (e.g. OSDI, Schirmer’s test and mean TBUT), was observed with gabapentin therapy in addition to topical therapies [80].

**Table 3:** Oral medications for corneal neuropathic pain with their dosage and mechanism of action [57].

Drug Class	Medication	Dosage	Mechanism of action
α2δ ligands [87]	Gabapentin	Gabapentin: start 100–300 mg nightly, escalate to 600–900 mg TID	Inhibits presynaptic Ca2+ channels, reduces release of excitatory neurotransmitters to suppress propagation of pain
	Pregabalin	Pregabalin: start 25–75 mg nightly, escalate to 150–200 mg BID	
Tricyclic anti-depressants (TCA) [88]	Amitriptyline Nortriptyline	Start at 10–25 mg nightly, escalate gradually up to 100 mg daily	Prevents monoaminergic neurotransmitter reuptake to inhibit pain transmission
SNRI [26]	Duloxetine	Start at 20 mg daily, escalate up to 60 mg daily	Same as for TCA
Anticonvulsant [26, 88]	Carbamazepine	Carbamazepine:	Believed to alter pro-inflammatory signalling mechanisms (e.g. GABA, glutamate), and exert a blockade on excitatory sodium channel activity and calcium channel influx
	Topiramate	Start at 100 mg BID, escalate up to 200–400 mg BID	
		Topiramate: Start at 25 mg BID and escalate weekly up to 200 mg BID within 6 weeks	
Opioid antagonist [88]	Naltrexone	1.5–2 mg nightly, escalate to 4–4.5 mg	Inhibits microglial cell activation in the CNS via TLR4 modulation to reduce production of inflammatory and excitatory molecules
Opioid agonist [26]	Tramadol	50 mg once or twice daily as needed	Weak μ-opioid receptor agonist (direct analgesia), downstream inhibits of noradrenaline and serotonin re-uptake (indirect analgesia)

Medication regimes should be tailored to individual patient needs and potential side-effects discussed.

*TCA* tricyclic antidepressant, *SNRI* serotonin-norepinephrine reuptake inhibitors, *TID* three times a day, *BID* twice a day, *CNS* central nervous system, *TLR* toll-like receptor.

Tricyclic anti-depressants (TCAs) have been utilised in the management of CNP and maybe classified as secondary amines (e.g. nortriptyline and desipramine) and tertiary amines (e.g. imipramine and amitryptline) [81]. TCAs inhibit noradrenaline uptake but may have effects via actions such as sodium channel blockade, sympathetic blockage, antagonism of N-methyl-D-aspartate glutamate receptors and anticholinergic activities [81, 82]. Due to a better safety profile, nortriptyline maybe preferred to tertiary amines, particularly in the elderly, as side effects such as confusion and postural hypotension maybe avoided. In neuropathic pain, the TCA can act in smaller doses and without needing to treat depression [81, 82]. In a retrospective study of 30 NCP patients, who had had an inadequate response to other systemic and topical treatments and with centralised component treated with nortriptyline on chart review there was a symptomatic improvement in CNP and mean quality of life scores also improved [53]. In this study, 33% of patients had more than 30% improvement in pain and 27% withdrew from treatment due to prolonged side-effects even though 22% improved [53]. Anti-convulsant/sodium channel blockers such as carbamazepine, oxcarbazepine or topiramate have also been used in CNP due to their ability to alter pro-inflammatory signalling and blockage of channels associated with nerve excitability[52, 83].

The selection of the most appropriate agent(s) is based on a patient’s needs and medical status with consideration of the agent that will be best tolerated and most effective [52]. To improve compliance with oral therapies patients should be counselled on their potential side effects and time course of action. In general, a low dose is trialled first and increased if needed noting that it may take 2–3 months for pain symptoms to improve, and that further improvement could take a year or more. If the response is partial an additional agent maybe added again starting at a low dose. Treatment is generally maintained for 2–3 years before weaning. At present, it cannot be predicted who will respond to which oral therapy such that treatments needed to be trialled [52].

### Adjuvant therapies

Limited clinical studies have reported on the use of adjuvant therapies in CNP (Table 4). In a small study of 12 veterans, some patients who had botulinium toxin A was administered to several sites on the forehead, reported decreased light sensitivity with a reduction in activity in brain areas that process pain seen on functional MRI [84]. The effects of botulinium toxin A are through a number of mechanisms that act via the trigeminal nerve pathway; with photophobia also improved via these pathways [84]. Both ocular surface disturbances such as dry eye and light can trigger these pathways [85], such that botulinium toxin maybe beneficial for CNP both with and without dry eye and with or without migraine. Trigeminal nerve stimulation over 6 months has been reported in a case series of veterans to decrease symptoms of ocular pain (pain intensity, light and wind sensitivity, burning sensation) particularly in those with a history of migraine [86]. Transcutaneous electrical stimulation and peri-ocular nerve blocks maybe used to reduce trafficking of pain signals to the central nervous system [52, 87, 88]. In patients with parasympathetic or sympathetic components underlying the pain sphenopalatine ganglion or superior cervical ganglion blocks may have a role in blocking nerve responses.

**Table 4:** Potential adjuvant therapies for corneal neuropathic pain [57].

Therapy	Dosage	Mechanism of action
Botulinum toxin [89]	35 units over 7 sites in forehead, adjusted as needed	Prevents release of pro-inflammatory substrates, blocks nociceptive signalling and inhibits ACh release at presynaptic nerve terminals, reducing muscle activity
Transcutaneous electrical stimulation [91]	Optimal usage not established; generally, a 20-min treatment nightly at least 3 sessions per week	Prevention of pain signalling via a combination of peripheral and central mechanisms
Peri-ocular nerve blocks [88]	Dosage should be individualised. Typically, a mixture of 3–4 mL of 0.5% bupivacaine mixed with 1 mL of 80 mg/mL methylprednisolone acetate and inject 0.5–1 mL is given around supraorbital, supra-trochlear, infraorbital nerves	Reversible block of sodium channels, involved in pain-related action potential trafficking into the CNS; suppresses ectopic firing on sensitised nerves
Parasympathetic ganglion block (sphenopalatine) and/or Sympathetic ganglion block (superior cervical) [92]	Various approaches (transnasal, transoral, supra/ infrazygomatic) for sphenopalatine; one study found pain control with delivery of 0.3 mL of 0.5% bupivacaine in patients with migraine; 1– 2 mL of 2–4% lidocaine via transnasal route in each nostril for pain. Direct image-guided blockade of superior cervical ganglion with local anaesthetic (bupivacaine 0.25%)	Reversal of parasympathetically mediated ocular vasodilation, increased permeability and release of pro-inflammatory mediators, causing sensitization of nociceptors. Superior cervical blockades suppress the sensitising sympathetic efferent drive to nociceptive nerves (in case of sympathetically maintained pain)

Data is needed to establish with certainty the dosages, efficacy, and safety.

*Ach* acetylcholine, *CNS* central nervous system.

### Surgical therapy

Case reports and case series suggest that refractory disease maybe managed with amniotic membrane transplantation (PROKERA, Bio-Tissue, Miami, FL) [89], corneal neurotization [90] or intranasal neurostimulation [91]. For such surgical approaches, robust data is needed on their safety and efficacy to support such approaches for use in routine clinical practice for CNP [52]. Clinical trials and registries may provide such data.

#### Considerations for practice

- Management of CNP management is generally multidisciplinary and may employ a range of treatments.
- Topical therapy may improve both symptoms of pain and the ocular surface, with success particularly in patients with peripheral neuropathic pain.
- Oral neuromodulators are often needed in central neuropathic pain when there is no response to topical therapy and are chosen based on individual patient needs.
- Measures to recognise and signpost support for mental health (cognitive behavioural therapy, emotional support and counselling) are often needed.

## Summary

### What is known about this topic

- Corneal neuropathic pain (CNP) is characterised by heightened experience of pain without corresponding clinical signs.
- Overlap with dry eye disease (DED) is not uncommon in patients with CNP.
- Peripheral CNP can be identified by a recovery with topical anaesthesia and generally resolves quickly whereas in central CNP there is often photophobia and management is more complex.

### What this study adds

- Knowledge of the underlying pathophysiology and clinical presentation of CNP can assist clinicians in diagnosing the condition.
- Clinicians are made aware of the need to consider CNP in patients with chronic dry eye disease (DED) that is refractory to treatment.
- Questionnaires can assist clinicians in identifying patients with CNP.
- The range of modalities used to manage for CNP should include mental health support if needed.
